# Environmental and Rhizosphere Microbiome Drivers of Metabolic Profiles in *Gastrodia elata*: An Integrative Analysis of Soil, Metabolomics and Anti-Inflammatory Readouts

**DOI:** 10.3390/foods14244265

**Published:** 2025-12-11

**Authors:** Yan Yang, Longxing Guo, Yongguo Li, Miaomiao Ji, Tingting He, Kaiming Hou, Jian Li, Haonan Zhang, Zhilong Shi, Haizhu Zhang

**Affiliations:** 1 College of Pharmacy, Dali University, Dali 671000, China; 17352095358@163.com (Y.Y.); 17787430793@163.com (Y.L.); 18872516236@163.com (M.J.); 17585256683@163.com (T.H.); h18288429185@163.com (K.H.); li178294@163.com (J.L.); 18747866574@163.com (H.Z.); 2 Yunnan Key Laboratory of Gastrodia and Fungi Symbiotic Biology, Zhaotong University, Zhaotong 657000, China; 3 Yunnan Western Yunnan Medicinal and Edible Plant Resources Development Engineering Research Center, Dali 671000, China; 4 College of Pharmacy, Nanjing University of Chinese Medicine, Nanjing 210023, China; guolongxin08@163.com

**Keywords:** rhizosphere microorganisms, medicine-food homologous, anti-inflammatory, metabolomics

## Abstract

Background: Gastrodiae Rhizoma, the dried tuber of *Gastrodia elata* Bl. (Orchidaceae), is a traditional Chinese medicinal (TCM) and edible plant. Its quality formation is closely associated with rhizosphere microorganisms; however, the specific underlying mechanisms remain unclear. Methods: Tubers and rhizosphere soils were collected from seven major production regions of *G. elata*. Soil physicochemical properties were analyzed, and integrative analyses combining soil microbiome and untargeted metabolome profiling were conducted. The anti-inflammatory activity of *G. elata* extracts was evaluated using a RAW264.7 macrophage model. Multivariate statistical approaches, including OPLS-DA and correlation network analysis, were used to decipher relationships among environmental factors, microbial communities, metabolic profiles, and bioactivities. Results: A total of 39,250 bacterial ASVs and 10,544 fungal ASVs were identified. The bacterial community, dominated by *Proteobacteria* and *Acidobacteria*, was strongly influenced by soil chemical factors, including pH and total nitrogen. The fungal community, primarily composed of *Ascomycota* and *Basidiomycota*, exhibited marked sensitivity to altitudinal gradients. Correlation analysis revealed that key secondary metabolites, including flavonoids and phenolic acids, along with their anti-inflammatory activities, were significantly associated with rhizosphere microorganisms such as *Edaphobaculum*, *Hypocrea*, and *Pseudomonas*. Conclusions: Our findings outline the pathways connecting environmental factors, the microbiome, and functional metabolites in *G. elata*, highlighting the importance of environmental–microbial interactions in determining metabolic outcomes. This work provides new insights into the ecological and molecular mechanisms behind the quality formation of this medicinal plant.

## 1. Introduction

Gastrodiae Rhizoma is the dried tuber of *G. elata* Bl., a plant belonging to the Orchidaceae family, first documented in *Shen Nong’s Classic of Materia Medica (Shennong Bencao Jing)* [1]. It is primarily distributed in Sichuan, Guangdong, Yunnan, and Guizhou provinces in China, with Yunnan recognized as an authentic (daodi) producing area, and possesses both traditional and modern values as a medicinal and edible plant [2]. Modern pharmacological research has demonstrated that it exhibits diverse effects, including anticonvulsant, antidepressant, antiepileptic, and anti-inflammatory activities [3,4,5,6,7,8]. In 2023, China’s National Health Commission officially recognized *G. elata* as a homologous plant for medicine and food, significantly expanding its application in functional foods, health drinks, dietary supplements, and providing new growth opportunities for the traditional Chinese medicine industry [9]. At present, more than 500 pharmaceutical formulations containing *G. elata* as the principal ingredient—such as gastrodin injection, *G. elata* capsules, and *G. elata* tablets—have been approved for clinical use by the National Medical Products Administration of China (NMPA) [10]. In addition, approximately 120 health products formulated with *G. elata* or its extracts have been developed [11]. These products are reported to exhibit multiple health-promoting effects, including improving sleep quality, assisting in blood pressure regulation, and modulating immune function. However, excessive harvesting and habitat destruction during the 1970s and 1980s led to a sharp decline in wild *G. elata* resources. Consequently, the species has been listed as a Class II nationally protected wild plant in China and classified as an endangered species [12]. Although artificial cultivation has become the main source of *G. elata*, significant differences in soil physicochemical properties and microbial community composition compared with its natural habitats have resulted in reduced levels of bioactive compounds and compromised medicinal quality, posing a major bottleneck for the sustainable and high-quality development of the *G. elata* industry.

Soil microorganisms, particularly mycorrhizal fungi and the broader rhizosphere microbiome, are increasingly recognized not merely as nutrient sources but as integral regulators of plant secondary metabolism, immune function, and overall performance. The rhizosphere constitutes a dynamic interface in which root exudates selectively recruit and structure microbial communities, while microbial metabolites, phytohormones, and other bioactive compounds reciprocally modulate plant physiology, shaping growth, defense, and the accumulation of specialized metabolites [13,14,15]. Plant-derived specialized metabolites, including flavonoids, coumarins, and triterpenes, selectively shape rhizosphere microbial assemblages by enriching beneficial taxa and constraining potential pathogens [16]. Conversely, the microbiome—comprising mycorrhizal fungi and diverse beneficial bacteria—generates phytohormones, volatile metabolites, antimicrobial compounds, and other signaling molecules that modulate plant immunity, secondary metabolism, and stress resilience [17]. This bidirectional exchange establishes a mechanistic framework in which plants modulate microbial community composition through root exudation, and microbes in turn adjust their functional outputs in ways that influence plant immunity, stress resilience, and the synthesis of specialized metabolites. Emerging evidence indicates that these interactions are highly dynamic: under abiotic stress, plants reconfigure their exudation profiles to recruit beneficial microbial assemblages that subsequently enhance stress tolerance [18,19,20]. *G. elata* is a completely mycoheterotrophic perennial plant [21], whose seed germination and growth rely heavily on symbiotic associations with fungi from the genera *Mycena* and *Armillaria* [22,23]. Ecological adaptation and reproduction depend on a unique ternary nutritional model involving *G. elata*, *Armillaria*, and saprophytic wood-decaying fungi [24]. Consequently, current artificial cultivation predominantly simulates the forest floor environment. Although industrial-scale production has been achieved, the plant’s long growth cycle and vulnerability to factors such as climate variation, pests, diseases, soil microbiome, and continuous cropping practices result in substantial uncertainty and unstable quality. These challenges have constrained the sustainable development of the *G. elata* industry, highlighting the urgent necessity for in-depth research into its symbiotic mechanisms and cultivation ecology.

Therefore, clarifying how *G. elata*, its symbiotic fungi, the surrounding soil microbiome, and soil physicochemical properties interact to influence pharmacological activity is essential for improving the stability and quality of cultivated plants. Although previous studies have linked microbial community composition and soil factors to *G. elata* quality, an integrated, mechanistic understanding remains lacking. To address this gap, we combined metagenomics, metabolomics, and analyses of soil physicochemical properties and environmental effects on pharmacological activity to tackle two core questions: (1) Which keystone microbial taxa underpin quality formation in *G. elata*? (2) Through which nutrient-cycling processes and signaling pathways do these microbes regulate secondary metabolism? We propose that (i) rhizosphere microbial assembly is a primary driver of bioactive compound accumulation, and (ii) specific symbiotic fungi serve as central hubs within the microbial interaction network. Elucidating these mechanisms will establish a mechanistic basis for improving *G. elata* quality and supporting sustainable cultivation practices.

## 2. Materials and Methods

### 2.1. Sample Collection and Processing

This study utilized rhizomes of *G. elata* collected from seven distinct geographical sources in December 2024. The collection sites were as follows: Tengchong City (MB); Ludian (DX), Yiliang (XCB), Yongshan (YS), and Zhenxiong (ZX) Counties in Zhaotong City; Luquan County (LQ) in Kunming City; and Hezhang County (HZ) in Guizhou Province (Table 1). All materials were sourced from standardized imitation-wild cultivation systems, in which *G. elata* was grown under forest or forest-edge environments that closely mimic its natural habitat. This approach ensured consistent ecological conditions and minimized site-specific variability among samples. All specimens were authenticated by Prof. Haizhu Zhang based on distinctive morphological characteristics following the taxonomic criteria of the Flora of China.

The researchers employed a five-point sampling method to randomly collect five *G. elata* tubers. After thorough washing and processing, the materials were pooled and prepared as three biological replicates. Rhizosphere soil samples were collected from within 10 mm of the tuber surface, whereas bulk soil samples were obtained from the 10–15 cm zone surrounding the root area. Each soil type was homogenized separately, and three biological replicates were generated for each category. Following collection, both soil and tuber samples were immediately stored at −80 °C.

### 2.2. Retrieval of Climate Data for Different Sampling Locations

The climate data corresponding to each sampling site were obtained from the China Meteorological Data Service Center (https://data.cma.cn/). The selected climatic variables included the annual maximum and minimum temperatures (MXAT and MIAT), annual mean temperature (MMT), mean monthly temperature difference (MMTD), annual precipitation (APP), annual average humidity (AAH), and altitude. These variables were chosen to represent the major climatic conditions that may influence the distribution and growth of *G. elata*. All data were extracted based on the geographical coordinates of each sampling site and averaged over the harvesting year of *G. elata* to minimize the influence of interannual variability (Table 1).

### 2.3. Analysis of Soil Physicochemical Properties

Soil pH was determined using the electrode method. Large soil particles were removed, and 25 g of soil was weighed and placed in a small beaker. Distilled water was added at a ratio of 1:1 (*w*/*w*) for air-dried soil (2:1 for acidic soils), thoroughly stirred, and left to stand for 30 min prior to measurement [25].

Soil organic matter (SOM) was determined using the dichromate oxidation method. Ammonium nitrogen (NH_4_^+^-N) was quantified by Nessler’s reagent colorimetry, hydrolyzed nitrogen by alkaline hydrolysis distillation, nitrate nitrogen (NO_3_^−^-N) by the nitric acid powder method, available phosphorus by molybdenum blue colorimetry, available potassium (AP) by the sodium tetraphenyl boron turbidimetric method, and total nitrogen (TN) by acid hydrolysis colorimetry [26].

### 2.4. Soil Microbiome Analysis

#### 2.4.1. Genomic DNA Extraction and PCR Amplification

Genomic DNA was extracted using the Cetyltrimethyl Ammonium Bromide (CTAB) method, and quality was assessed by agarose gel electrophoresis. DNA was then diluted to a concentration of 1 ng/μL and used as a PCR template. PCR amplification was performed with barcode-tagged primers using Phusion^®^ High-Fidelity PCR Master Mix (NEB). Bacterial diversity was analyzed by amplifying the 16S rRNA V4 region (515F/806R primers), and fungal diversity was assessed using the ITS region (ITS5-1737F/ITS2-2043R primers).

#### 2.4.2. PCR Product Purification, Library Construction, and Sequencing

PCR products were first examined by 2% agarose gel electrophoresis, and qualified amplicons were purified using magnetic beads, quantified by enzyme labeling, and mixed in equal amounts according to their concentrations. After thorough mixing, the pooled PCR products were re-examined by 2% agarose gel electrophoresis, and target bands were recovered using a Qiagen gel extraction kit (Qiagen, Germantown, MD, USA). The purified products were then used for library construction with the NEBNext Ultra II DNA Library Prep Kit (E7645B, NEB, Ipswich, MA, USA). The resulting libraries were quantified using Qubit and qPCR, and, after passing quality control, were sequenced on a NovaSeq 6000 platform (Illumina Company, San Diego, CA, USA).

### 2.5. Non-Targeted Metabolomics Analysis

#### 2.5.1. Equipment and Reagents

Mass spectrometer (TripleTOF 6600, SCIEX, Framingham, MA, USA), ultra-high-performance liquid chromatograph (LC-30A, Shimadzu, Kyoto, Japan), Centrifuge (5424R, Hamburg, Germany, Eppendorf).

Methanol (67-56-1, Chromatography Grade, Merck, Darmstadt, Germany), Methanol (67-56-1, Chromatography Grade, Merck), Acetic Acid (64-19-7, Chromatography Grade, Rohn, Philadelphia, PA, USA), Ammonium formate (540-69-2, 469 Chromatography grade, Aladdin, Shanghai, China), Ammonia solution (1336-21-6, Chromatography grade, Aladdin), Formic acid (64-18-6, Chromatography grade, Aladdin), Internal standard: 2-Chloro-L-phenylalanine (purity: 98%, Manufacturer: Bailingwei Batch No.: 106151, CAS: 103616-89-3, Internal standard concentration: 1PPM (mg/L)).

#### 2.5.2. Sample Preparation and Extraction

Samples were freeze-dried (Scientz-100F, SCIENTZ, Zhejiang, China) and ground (MM 400, 30 Hz, 1.5 min). Fifty milligrams of powdered sample were weighed and extracted with 1.2 mL of pre-cooled (−20 °C) 70% methanol solution containing internal standards. Extraction was performed by intermittent oscillation (6 × 30 s). After centrifugation (12,000 rpm, 3 min), supernatants were collected, filtered through a 0.22 μm microporous membrane, and transferred to injection vials for UPLC-MS/MS analysis.

#### 2.5.3. HPLC Conditions

Samples were analyzed using two LC/MS methods. For positive ion mode analysis, samples were separated on a Waters ACQUITY Premier HSS T3 (1.8 µm, 2.1 × 100 mm) column with a mobile phase consisting of 0.1% formic acid-water (A) and 0.1% formic acid-acetonitrile (B). The gradient conditions were as follows: 5–20% B (0–2 min), 20–60% B (2–5 min), 60–99% B (5–6 min), maintained for 1.5 min, returned to 5% B within 0.1 min, and equilibrated for 2.4 min. Analytical conditions included a column temperature of 40 °C, flow rate of 0.4 mL/min, and injection volume of 4 μL. Negative ion mode analysis employed identical conditions.

#### 2.5.4. Mass Spectrometry (MS) Conditions (AB)

Data were acquired using Information-Dependent Acquisition (IDA) mode controlled by Analyst TF 1.7.1 software. Ion source parameters were set as follows: GAS1 and GAS2 at 50 psi, curtain gas (CUR) at 25 psi, temperature at 550 °C, declustering potential (DP) at ±60 V, and ion spray voltage (ISVF) at ±5000 V. The time-of-flight mass spectrometry (TOF MS) scanning range was 50–1000 Da with an accumulation time of 200 ms and enabled dynamic background subtraction. Product ion scanning ranged from 25 to 1000 Da, with an accumulation time of 40 ms, collision energy of ±30 V, collision energy diffusion of 15, resolution set to UNIT, charge state at 1, intensity threshold at 100 CPS, isotopes within 4 Da excluded, mass tolerance at 50 ppm, and 18 candidate ions monitored per cycle.

#### 2.5.5. Quality Control and Data Processing

This study employed a non-targeted metabolomics strategy, using LC–MS peak intensities for the relative quantification of metabolite abundances to enable comparative analysis of metabolic variation among samples. To assess system stability and reproducibility, a pooled quality control (QC) sample was prepared by combining equal aliquots of all extracts and analyzed at regular intervals (every ten injections) (Figure S5). Raw MS data were converted to mzML format using ProteoWizard and processed in XCMS for peak detection, alignment, and retention time correction. Features with more than 50% missing values within any group were removed, and remaining missing values were imputed using the k-nearest neighbors (KNN) algorithm. Peak areas were normalized and batch effects corrected using support vector regression (SVR). Metabolite identification was performed by matching the processed spectra against the in-house MWDB (MetWare Database) and public databases including METLIN (https://metlin.scripps.edu/index.php), HMDB (Human Metabolome Database) (https://hmdb.ca/) and KEGG (https://www.kegg.jp/), MoNA (MassBank of North America) (https://mona.fiehnlab.ucdavis.edu/) and MassBank (http://www.massbank.jp/) supplemented with machine-learning–based predicted spectra. Only metabolites with an identification score > 0.5 and a coefficient of variation (CV) < 0.5 in QC samples were retained. Data from positive and negative ionization modes were then integrated by selecting the features with the highest annotation confidence and lowest CV, yielding the final dataset for downstream analyses.

### 2.6. Biological Potency Assays

#### 2.6.1. Determination of RAW264.7 Cell Viability Using the CCK-8 Assay

Mouse monocyte–macrophage RAW264.7 (CL-0190, Pricella, Shanghai, China) were cultured at 37 °C in a humidified atmosphere containing 5% CO_2_. The cells were seeded into 96-well plates at a density of 1 × 10^4^ cells/well and incubated for 24 h. Subsequently, the culture medium was replaced with fresh medium containing various concentrations of *G. elata* extracts (0, 0.0125, 0.025, 0.05, 0.1, 0.2, 0.4, 0.8, and 1.6 mg/mL), and the cells were further incubated for another 24 h. Cell viability was then determined using the Cell Counting Kit-8 (BS350A, biosharp) assay according to the manufacturer’s instructions.

#### 2.6.2. The Anti-Inflammatory Effects of *G. elata* Extracts on RAW264.7 Cells Were Evaluated Using Enzyme-Linked Immunosorbent Assay (ELISA) Kits

When RAW264.7 cells reached approximately 80% confluence, cells were seeded into 6-well plates at approximately 1 × 10^6^ cells/well and cultured for 24 h. Cells were divided into groups: normal control (DMEM only), model group (1 μg/mL LPS + 25 ng/mL IFN-γ), treatment groups (low 0.1, medium 0.5, high 1 mg/mL CBE), and a positive control (15 μg/mL celecoxib) [27]. After an additional 24 h of incubation, culture supernatants were collected and stored at −80 °C. Levels of IL-6, NO and TNF-α were determined by double-antibody sandwich ELISA (mlbio, 20241117220A), and concentrations were calculated using standard curves. Three independent biological replicates were performed for each experimental group to ensure the reliability and reproducibility of the results.

### 2.7. Data Statistical Analysis

Raw soil microbial sequencing data were initially filtered using fastp (v0.22.0) to obtain high-quality reads. Paired-end sequences were then merged using FLASH (v1.2.11) to generate clean tags. Subsequently, chimeric sequences were identified and removed using VSearch (v2.22.1) by comparison with the reference database. Species annotation of ASVs was conducted using Mothur (v1.48) against the SILVA SSU rRNA database [28]. Analysis of species abundance and beta diversity was performed in R (v4.2.0) using the phyloseq (v1.40.0) and vegan (v2.6.2) packages. Community differences were analyzed with LEfSe (v1.1.2), while ANOSIM analysis was conducted using the vegan package. PCA, Pearson correlation analysis, and OPLS-DA of metabolomics data were executed using R (v4.1.2) and MetaboAnalystR (v1.0.1). Statistical analyses of soil physicochemical properties were performed using one-way analysis of variance (ANOVA) in SPSS (v26.0).

## 3. Results

### 3.1. Soil Microbial Diversity and Community Differentiation

**Figure 1:**
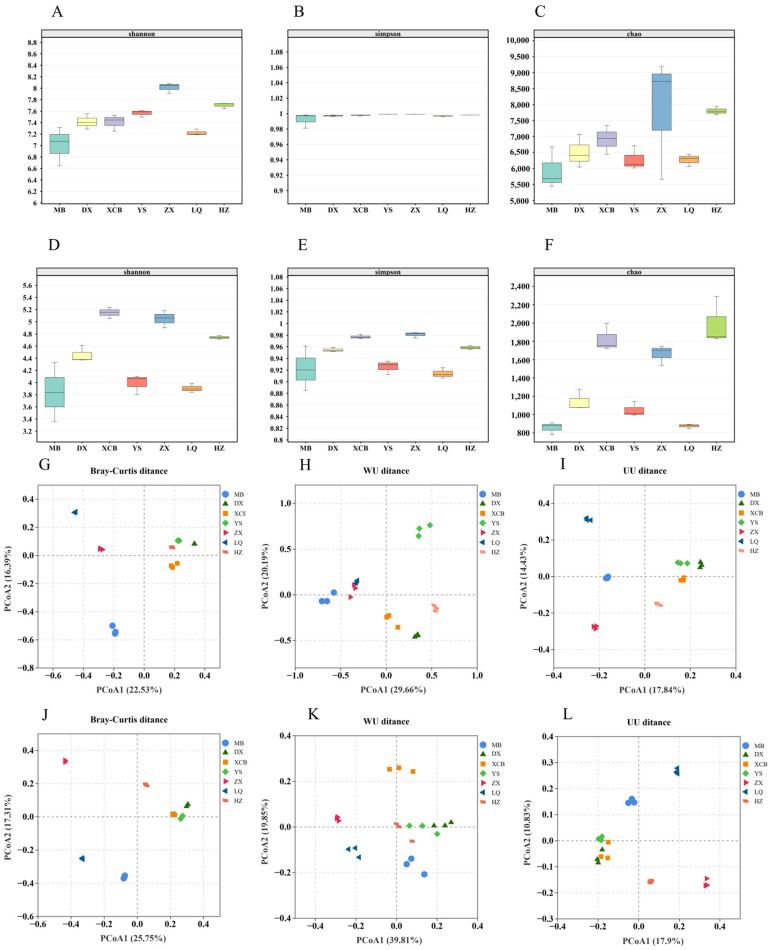
α-diversity and β-diversity of microbial community. The A-F plots represent the Chao (**A**), Shannon (**B**) and Simpson (**C**) α diversity indices of the bacterial community, and the Chao (**D**), Shannon (**E**) and Simpson (**F**) α diversity indices of the fungal community, respectively. Different letters in the figure indicated significant differences (*p* < 0.05). The β diversity of bacterial community based on Bray–Curtis (**G**), WU (**H**) and UU distance (**I**), and the β diversity of fungal community based on Bray–Curtis (**J**), WU (**K**) and UU distance (**L**) were shown in the G-L diagram, respectively.

High-quality sequences obtained from each sample were denoised to generate amplicon sequence variants (ASVs), and representative sequences were subsequently assigned to taxonomic classifications. In total, 2,130,414 high-quality 16S rRNA gene reads (average length: 253 bp) and 2,150,102 high-quality ITS2 reads (average length: 231 bp) were retained after quality filtering. Denoising produced 39,250 bacterial ASVs across rhizosphere soils from the seven planting regions, with ZX exhibiting the greatest richness (8936 ASVs) and MB the lowest (4435 ASVs). For fungi, 10,544 ASVs were identified, with the highest richness observed in HZ (1839 ASVs) and the lowest in YS (767 ASVs). α-Diversity analyses revealed clear spatial heterogeneity in microbial communities among planting regions. The bacterial Shannon index differed significantly across areas (*p* < 0.001), following the trend ZX > YS > XCB > MB > LQ > HZ > DX (Figure 1A). In contrast, both Simpson and Chao1 indices showed no significant differences (*p* > 0.05; Figure 1B,C), indicating comparable bacterial evenness and estimated richness across sites. For fungi, all three α-diversity indices—Shannon, Simpson, and Chao1—varied significantly among regions (*p* < 0.001; Figure 1D–F).

β-diversity analysis based on Bray–Curtis (BC), weighted UniFrac (WU), and unweighted UniFrac (UU) distances (Figure 1) demonstrated significant differences in bacterial and fungal community structures across planting areas. Specifically, the bacterial BC and UU analyses revealed significant separation among all seven areas (Figure 1G,I), while the WU analysis indicated partial overlap between LQ and ZX communities (Figure 1H). In fungal communities, the BC analysis showed significant separation of all planting areas (Figure 1J), whereas the WU analysis indicated partial overlap between YS and DX (Figure 1K), and the UU analysis showed partial overlap among YS, XCB, and DX (Figure 1L).

### 3.2. Soil Microbial Community Composition and Dominant Taxa

**Figure 2:**
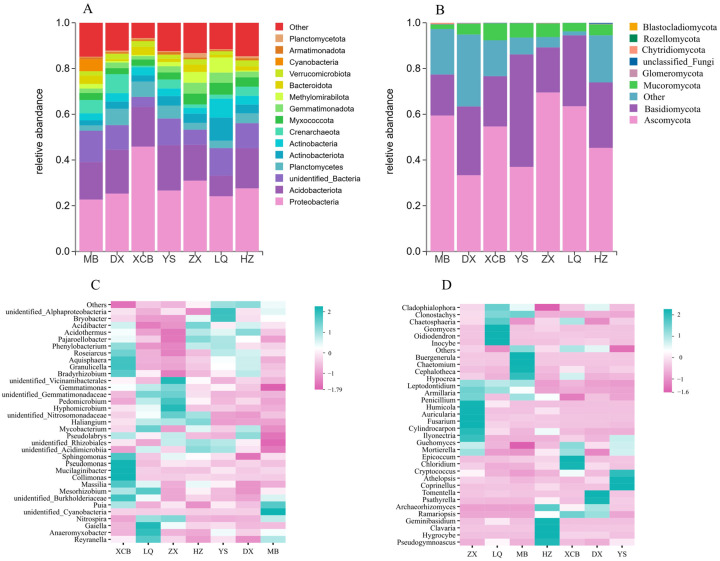
The most abundant phylum-level bacteria (**A**) and fungi (**B**), as well as the distribution of the top 35 bacterial (**C**) and fungal (**D**) genera, are shown in the figure.

At the phylum level (Figure 2), the bacterial community was predominantly composed of *Proteobacteria*, with relative abundances ranging from 22.7% to 45.8%. Additionally, *Acidobacteria* (9–19.8%) and *Planctomycetes* (2.3–6.7%) also exhibited relatively high abundance. The fungal community was primarily dominated by *Ascomycota* (37–69.5%), *Basidiomycota* (18–49.2%), *Mucoromycota* (1.9–7.4%), and other minor phyla. Soil microbial communities from the rhizosphere of *G. elata* displayed significant variation among different geographical origins at the phylum level. The relative abundances of *Proteobacteria* and *Acidobacteria* in bacterial communities (Figure 2A) and *Ascomycota* and *Basidiomycota* in fungal communities (Figure 2B) each exceeded 10%. Notably, the XCB area exhibited the highest relative abundance of *Proteobacteria* (45.8%), the ZX planting area showed the highest abundance of *Ascomycota* (69.5%), and the YS planting area had the highest relative abundance of *Basidiomycota* (49.2%), significantly exceeding other areas. At the genus level (Figure 2C,D), the dominant bacterial and fungal genera in rhizosphere soil showed pronounced spatial heterogeneity. Dominant bacterial genera included *Acidobacterium*, *Bradyrhizobium*, and *Sphingomonas*, whereas fungal genera exhibited clear regional specificity. Specifically, Humicola dominated in HZ, *Mortierella* was predominant in XCB, *Trechispora* was most abundant in DX, and *Penicillium* was dominant in ZX.

**Figure 3:**
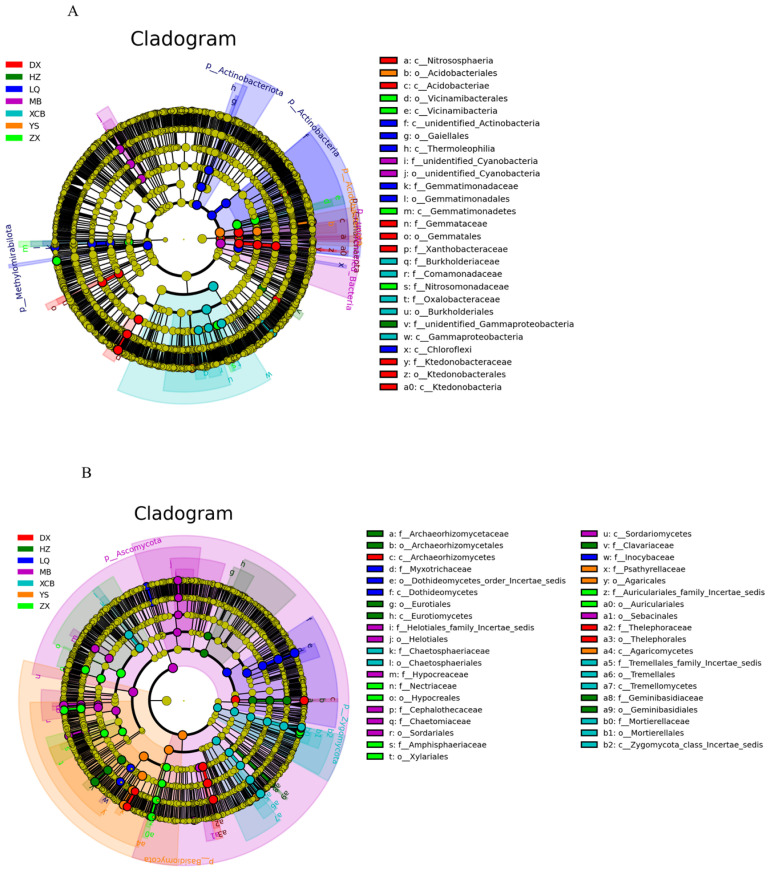
LEfSe algorithm screened out fungi (**A**) and bacteria (**B**) with significant differences ([LDA] scores > 4.3 and *p* < 0.05) at multiple classification levels of microbial communities in seven regions. The color dots represent the taxonomic units with significant differences in abundance between different sampling points, which represent the five levels of boundary, phylum, class, order, family and genus from the center to the outside.

The LEfSe analysis (Figure 3) was performed to identify significantly different abundance characteristics among rhizosphere soils from various locations. Biomarkers classifying microbial communities at the family level were determined based on strict criteria (linear discriminant analysis [LDA] scores > 4.3 and *p* < 0.05). A total of 27 biomarkers were identified in bacterial communities, distributed across different *phylogenetic* groups including *Crenarchaeota*, *Acidobacteria*, and *Actinobacteria.* Additionally, 39 microbial markers were identified in fungal communities, covering 7 classes, 15 orders, and 17 families.

### 3.3. Environmental Drivers Shaping Soil Microbial Community Assembly

Soil physicochemical properties including pH, SOM, TN, TP, TK, AP, AK, NO_3_^−^-N, and NH_4_^+^-N showed significant variation among the seven planting areas (*p* < 0.01, Table 2). The XCB and HZ areas exhibited the highest SOM and TN content, while ZX was lowest. The differences in TN content were not substantial, although MB was slightly higher than other areas. The LQ area had the highest TK content, XCB was notably enriched in AK, and DX showed significantly higher AP and NH_4_^+^-N, NO_3_^−^-N levels compared with other locations. In terms of pH, the ZX area exhibited a near-neutral condition suitable for most plants, whereas YS and DX had the most acidic soils.

**Figure 4:**
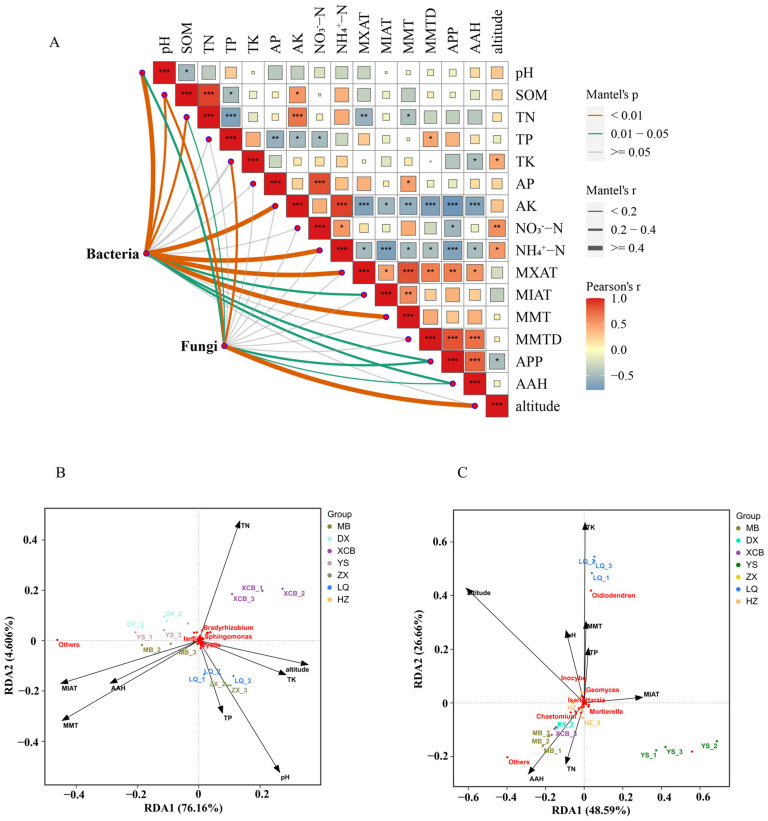
Mentel test (**A**) and RDA measured the relationship between soil microbial community composition and soil physical and chemical properties of bacteria (**B**) and fungi (**C**). * < 0.05; ** < 0.01; and *** *p* < 0.001.

The Mantel test results (Figure 4) indicated significant correlations between rhizosphere microbial community structures and various environmental factors. Bacterial community composition showed significant correlations with pH, SOM, AK, NH_4_^+^-N, MXAT, and MMT (Mantel’s *p* < 0.01, r = 0.2–0.4). In contrast, fungal community composition correlated significantly with SOM, T, altitude, and APP. Notably, pH had a strong explanatory influence on both bacterial and fungal communities, while the effects of climate and altitude were relatively weaker. Therefore, pH, SOM, and TN were key environmental factors driving shifts in the microbial community structure of *G. elata*. Further redundancy analysis (RDA, Figure 4) revealed the dominant roles of environmental variables in microbial community variation. For bacterial communities, the first RDA axis (RDA1) explained 76.16% of the variation, primarily driven by pH and TN, with significant contributions from TP, TK, and altitude. Conversely, fungal community variation (RDA1, 48.59%) was primarily influenced by pH and altitude, followed by TN and TP. Notably, all sampling sites were located within subtropical mountainous regions between 24° and 28° N latitude, with limited latitudinal range; hence, diversity differences were predominantly shaped by altitude gradients and local environmental conditions. From a longitudinal perspective, sampling sites concentrated in the northeastern Yunnan-western Guizhou border zone (98–105° E), characterized by complex terrain and intersecting canyons. Community diversity was lowest in western Tengchong (98°, 1493 m). Most central Zhaotong sites (103–105°, including YS, ZX, DX, XCB) exhibited moderate to high diversity levels. The eastern site, HZ (105°, 1659 m), had moderate altitude and showed the highest microbial abundance and diversity. Generally, bacterial communities depended more strongly on soil nutrient parameters (pH, TN), whereas fungal communities showed greater sensitivity to geographical factors, especially altitude.

### 3.4. Geographical Discrimination of G. elata Based on Metabolite Profiling

**Figure 5:**
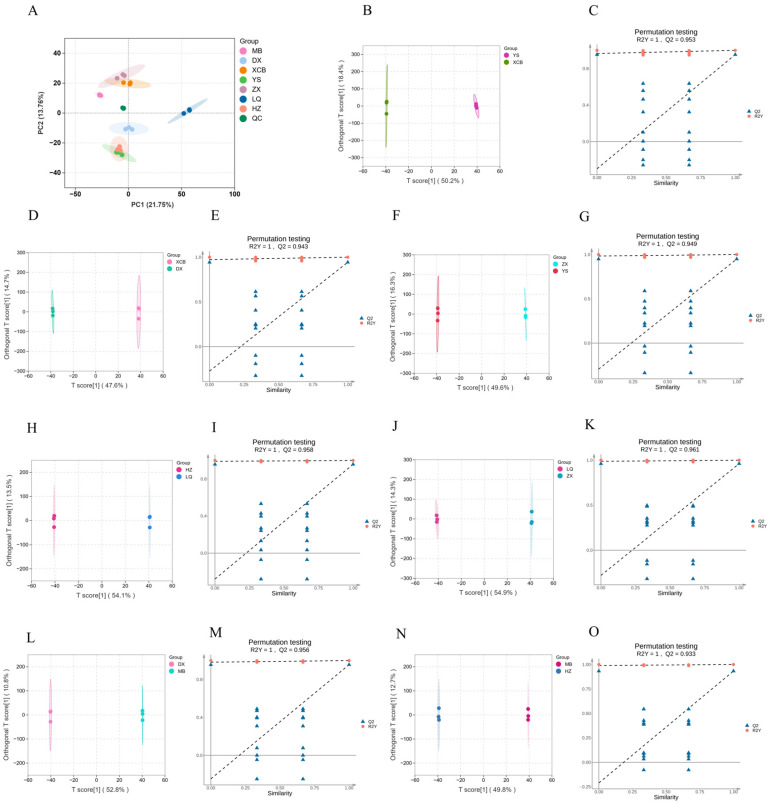
Metabolomics of *Gastrodia elata* tubers from seven producing areas and PCA score of QC samples (**A**). OPLS-DA analysis and permutation test ((**B**,**C**) YS vs. XCB; (**D**,**E**) XCB vs. DX; (**F**,**G**) ZX vs. YS; (**H**,**I**) HZ vs. LQ; (**J**,**K**) LQ vs. ZX; (**L**,**M)** DX vs. MB; (**N**,**O**) MB vs. HZ)).

Plant secondary metabolites, owing to their unique chemical structures and diverse bioactivities, exhibit significant potential applications in food additives, functional foods, and novel drug development. This study systematically conducted an untargeted metabolomic analysis of *G. elata* samples from seven distinct regions. A total of 2218 metabolites (positive ion mode) and 1471 metabolites (negative ion mode) were identified and classified into 20 compound classes. Composition analysis revealed that amino acids and derivatives (37.52%) predominated, followed by benzene rings and substituted derivatives (8.76%) and organic acids (8.76%). Alkaloids (6.01%) were notably abundant, and there was substantial presence of antioxidant compounds, including flavonoids (3.03%) and phenolic acids (3.93%). Additionally, terpenes (1.68%), lignans, and coumarins (1.19%) were present at lower levels, while trace constituents such as tannins (0.04%) and quinones (0.2%) may play crucial roles in plant defense mechanisms (Figure S1, Table S1). According to principal component analysis (Figure 5A), significant metabolite composition differences were observed among MB, ZX, XCB, LQ, and DX sites. The first principal component (PC1) accounted for 21.75% of total variation, while PC2 explained 13.76%. Further analysis using an OPLS-DA model demonstrated excellent model reliability (R2Y > 0.999, Q2 > 0.95) across seven pairwise comparisons (Figure 5B–O). Permutation validation (Q2 intercept < 0) confirmed the absence of model overfitting. Analysis of differential metabolites showed significant metabolic variation across planting areas. The greatest difference was observed between LQ and ZX (1014 metabolites), whereas MB and YS (556 metabolites) exhibited the least variation. The number of differential metabolites in other comparisons was as follows: HZ vs. LQ (905), DX vs. MB (727), YS vs. XCB (686), ZX vs. YS (681), and XCB vs. DX (618) (Figure S2). These findings not only elucidate the metabolic diversity of *G. elata* across different geographical origins but also provide a scientific foundation for future studies on bioactive compounds and geographical traceability.

### 3.5. Microbe–Metabolite Co-Functional Modules Associated with Food Functionality

**Figure 6:**
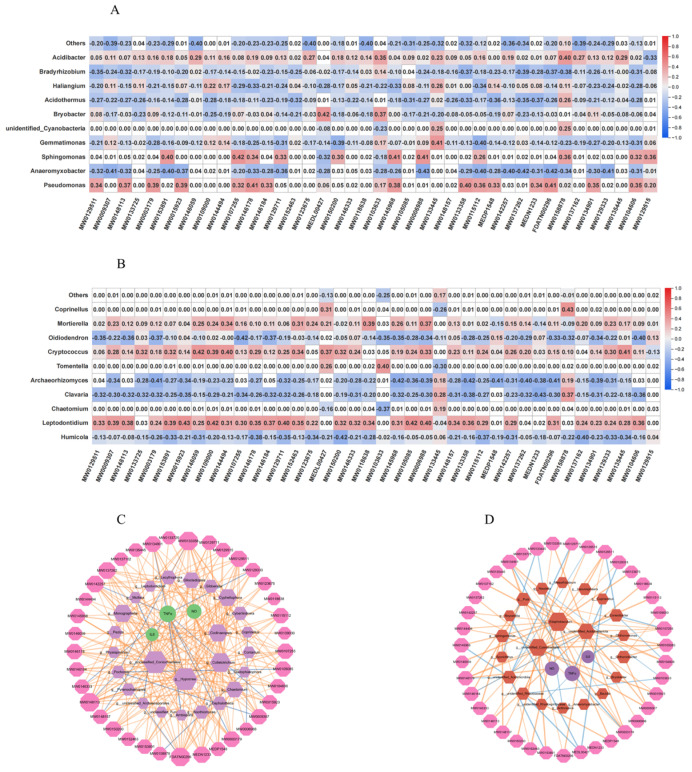
Statistical correlation analysis of differential metabolites and bacterial (**A**) and fungal (**B**) microbial communities in seven producing areas of *Gastrodia elata*. Triple correlation analysis of differential metabolism and anti-inflammatory activity and soil microbial community. The colour of the figures within the grid reflects the magnitude of correlation: red indicates positive correlation, blue indicates negative correlation. (**C**) Correlation between fungal community and differential metabolites and anti-inflammatory activity; (**D**) correlation between bacterial community and differential metabolites and anti-inflammatory activity.

Clarifying the relationship between microorganisms and their metabolic processes provides an important theoretical basis for scientifically regulating the quality formation and efficient cultivation of *G. elata* from different regions. This study identified core bacterial and fungal functional modules through correlation analyses between microbes and metabolites. Regarding bacteria (Figure 6A), the *unidentified_Cyanobacteria* module enhanced antioxidant and functional metabolite synthesis in *G. elata* by promoting the accumulation of 4-methylobellaryl acetate (r = 0.40), ribonolactone, and 2-methoxyhydroxyquinolone (r = 0.39), while inhibiting 2-hydroxychalcone production. Aromatic degradation modules (*Pseudomonas* and *Sphingomonas*) promoted flavonoid accumulation, including 8-benzodihydropyran trimer (r = 0.42) and 6-oxoheterocyclic flavonoids (r = 0.41), thereby reducing adverse metabolites. The anaerobic nitrogen-cycling module (*Anaeromyxobacter*, *Bradyrhizobium*) optimized nitrogen allocation, positively regulating metabolites such as O-phospho-L-tyrosine (r = 0.39) and nicotinic acid mononucleotide (r = 0.40), while negatively influencing dihydroquercetin (r = −0.43) and 3-methoxytyrosine synthesis (r= −0.37), thus enhancing amino acid and coenzyme metabolism and increasing active component proportions. Among fungi (Figure 6B), *Mortierella* markedly increased flavonoid synthesis, including 6-oxoheterocyclic flavonoids (r = 0.40) and 8-benzodihydropyran trimer (r = 0.39), while inhibiting 2-methoxyhydroquinone (r = −0.35). *Chaetomium* and *Humicola* synergistically regulated nitrogen metabolism, promoting beneficial amino acids and coenzymes. *Cryptococcus* exhibited a light-dependent metabolic profile, positively influencing ribolactone and umbelliferone derivative accumulation and negatively affecting 2-hydroxy ketone synthesis (r = −0.42). Thus, distinct microbial modules shaped the quality of *G. elata* by modulating metabolite accumulation, providing a novel theoretical basis for quality control and optimized cultivation.

### 3.6. Interaction Network of Microorganisms, Metabolites, and Anti-Inflammatory Activity

The integrative analysis revealed significant correlations among microbial taxa (16S), metabolites, and inflammatory factors (Figure 6C and Figure S4). Notably, the genus *unidentified_Rhodospirillaceae* exhibited a strong negative correlation with TNF-α (r = −0.964, *p* = 0.00045), while *Edaphobaculum* was also inversely correlated with TNF-α (r = −0.937, *p* = 0.00185). Conversely, *Puia* demonstrated a positive correlation with NO production (r = 0.893, *p* = 0.0068). Among metabolites, 2-methoxyhydroquinone showed a significant negative correlation with TNF-α (r =−0.857, *p* = 0.0137). Furthermore, robust associations were observed between metabolites and specific bacterial taxa; for example, 1-(2,6-dihydroxy-4-methoxyphenyl)-3-(4-methoxyphenyl) propan-1-one was positively correlated with unidentified_Cyanobacteria (r = 0.991, *p* = 1.46 × 10^−05^). Strikingly, *Edaphobaculum* emerged as a key taxon, positively correlated with multiple anti-inflammatory metabolites (e.g., 2-methoxyhydroquinone, r = 0.955, *p* = 0.0008) and negatively associated with TNF-α, suggesting its potential role in mitigating inflammation via modulation of metabolite production.

ITS analysis revealed that fungal taxa significantly regulate host inflammatory responses and metabolic networks (Figure 6D and Figure S4). The key fungal genus *Hypocrea* exhibited dual regulatory functions: it was significantly negatively correlated with TNF-α (r = −0.857, *p* = 0.0137) and positively correlated with metabolites such as 2-methoxyhydroquinone (r = 1, *p* < 0.001) and the flavonoid metabolite 6-(3,4-dihydroxy-6-methyl-5-oxohexan-2-yl)-5,7-dihydroxy-2-phenylchromene-4-one (r = 1, *p* < 0.001), indicating that it may exert anti-inflammatory effects via these metabolites. *Cyphellophora*, acting as a metabolic hub, showed a strong positive correlation with 4-hydroxyphenylacetylglycine (r = 0.964, *p* = 0.00045) and co-enrichment with 26 anti-inflammatory peptides (e.g., Cys Tyr, r = 0.893, *p* = 0.0068). Notably, *Codinaeopsis* demonstrated a significant positive correlation with L-asparaginyl-L-asparagine (r = 0.964, *p* = 0.00048), whereas *Ambispora* displayed a strong negative correlation with TNF-α (r = −0.927, *p* = 0.0027). Further metabolite-inflammation associations confirmed the anti-inflammatory potential of 2-methoxyhydroquinone (r = −0.857, *p* = 0.0137) and artemisinin (r = −0.821, *p* = 0.0234), thus establishing a comprehensive fungal-metabolite-anti-inflammatory regulatory network.

## 4. Discussion

### 4.1. Environmental Gradients Shaping Rhizosphere Microbial Community Differentiation

Through alpha and beta diversity analyses, we identified core functional microbial communities in the rhizosphere of *G. elata*, predominantly including *Proteobacteria*, *Acidobacteria*, and *Ascomycota*. Consistent with these findings, earlier studies reported these microbial taxa as dominant in rhizosphere communities of various plants such as *Chinese cabbage*, *Chinese fir*, and *Polygonatum sibiricu* [29,30,31]. Significant regional differences in dominant microbial community composition were also observed. For example, higher abundances of *Proteobacteria* in certain regions promoted root metabolism and defense responses, while shifts in the ratio of *Acidobacteria* to *Ascomycota* influenced interactions between *G. elata* and symbiotic fungi [32,33]. These microbial ecological differences not only shape the nutrient environment of the rhizosphere but also regulate the nutritional quality and pharmacological activity of *G. elata* by influencing its symbiotic system. At the genus level, bacterial and fungal communities in the rhizosphere soil exhibit significant spatial heterogeneity, reflecting sensitive responses to environmental conditions. Dominant bacterial genera, including *Acidobacterium*, *Bradyrhizobium*, and *Sphingomonas*, are widely distributed in various soils and recognized as core taxa involved in carbon and nitrogen cycling and plant growth promotion [34]. In contrast, fungal communities display strong locality specificity. For instance, *Humicola* in HZ, *Mortierella* in XCB, *Trechispora* in DX, and *Penicillium* in ZX are, respectively, associated with organic matter decomposition, nutrient cycling, lignin degradation, and antagonistic activities [35]. This coexistence pattern of a “bacterial core” and “fungal locality specificity” indicates that host selection and environmental conditions jointly drive structural differentiation of the rhizosphere communities [36].

Mantel tests and redundancy analyses (RDA) identified key environmental factors influencing the microbial community composition of *G. elata*. The results indicated that bacterial communities were highly dependent on the soil physicochemical parameters pH and TN, aligning with the consensus that soil pH largely governs global microbial diversity patterns and highlighting the specific role of nitrogen-cycling factors [37,38]. Furthermore, bacterial communities exhibited greater dependence on pH and nitrogen (TN) compared to fungal communities, whereas fungi demonstrated unique sensitivity to altitude gradients. This finding challenges the conventional paradigm that climate universally shapes microbial community patterns [39]. In highly heterogeneous mountain ecosystems, altitude gradients exert habitat-filtering effects beyond climatic scales by altering microtopographic temperature and moisture combinations at scales smaller than 100 m [40]. Fungi preferentially respond to microenvironmental gradients due to their greater phenotypic plasticity and dispersal capacity [41]. Conversely, bacteria remain strongly constrained by chemical parameters such as pH and TN due to biochemical limitations [42]. This “differential response of microbial communities at dual environmental boundaries” expands rhizosphere microecological theory, indicating that bacterial community assembly in acidic soils is primarily influenced by chemical factors, while fungal community structure is synergistically shaped by geographical and climatic factors.

### 4.2. Microbially Mediated Remodeling of the Metabolic Network in Quality Formation

Plant specialized metabolites can recruit and shape root-associated microbiota [43]. However, recent systems ecology studies have demonstrated that the relationship between small-molecule metabolites and rhizosphere microorganisms is fundamentally bidirectional [44], and that microbial metabolic activities, nutrient competition, and microbe–microbe interaction networks are equally critical determinants of community stability and functional output [45]. For example, Wang et al. [46] analyzed rhizosphere microbiota across 108 plant species and showed that small-molecule metabolite profiles can drive marked differences in microbial community assembly, selectively enriching certain taxa such as Rhizobium in response to metabolite availability, thereby reshaping the rhizosphere chemical landscape. In addition, ginseng-associated microbiota primarily influence saponin diversity [40], whereas the microbiota of Astragalus modulate flavonoid and astragaloside biosynthesis and those of Salvia miltiorrhiza are tightly linked to tanshinone and salvianolic acid production [47]. Collectively, these findings support a more microbe-centric ecological model, in which plant metabolites provide essential substrates, but the ultimate metabolic expression, compound transformation, and homeostatic maintenance depend largely on the architecture and equilibrium of rhizosphere microbial networks. Within this conceptual framework, our study proposes that the quality formation of *G. elata* is connected to these microbial networks, particularly through a multidimensional coupling system linking the metabolome, microbiome, and pharmacological activities. Our findings demonstrate that rhizosphere bacteria of *G. elata* precisely regulate metabolic pathways via three functional axes: the photosynthesis–photometabolism axis (Cyanobacteria → 4-methylobellaryl acetate), the aromatic degradation–flavonoid synthesis axis (*Pseudomonas*/*Sphingomonas* → 6-oxoheterocyclic flavonoids), and the anaerobic nitrogen–amino acid metabolism axis (*Anaeromyxobacter/Bradyrhizobium* → O-phospho-L-tyrosine). These axes finely modulate plant metabolic pathways, representing a functional coupling between microbial communities and host metabolism in *G. elata*, aligning closely with Bahram’s modular theory of microbial habitat adaptation [48]. Notably, this study highlights that these functional axes are not merely positively regulated; rather, negative regulatory interactions also contribute significantly to maintaining metabolic homeostasis, complementing traditional views focused solely on positive interactions [49]. *Mortierella*, identified as a core effector in flavonoid transformation, acts independently of *mycorrhizal* symbiosis [50], demonstrating that non-symbiotic fungi can directly regulate medicinal component biosynthesis. Concurrently, the nitrogen metabolism balance network involving *Chaetomium* revealed a fungal ecological strategy to optimize plant resource allocation via a metabolic “brake” mechanism [51]. Furthermore, the *Cryptococcus*-mediated light-responsive metabolic axis significantly corresponded with altitude gradients, supporting the theory that geographical and climatic factors at the microecological scale drive microbial regulation of plant metabolism [52,53]. Together, these findings reveal that rhizosphere microbial network equilibrium, rather than individual taxa or single pathways, underlies the metabolic stability and quality formation of *G. elata*.

### 4.3. Integrated Mechanisms Underlying the Microbe-Metabolite-Pharmacology Interplay

A bidirectional regulatory relationship exists between plant innate immunity and microbial communities; microbes are both targets of immune regulation and modulators of immune homeostasis via metabolic products and signaling molecules [54]. Recent studies have shown that plant immunity is not solely a passive defense mechanism but actively shapes rhizosphere microbial communities through chemical and signaling pathways [55]. For example, leaf-derived salicylic acid signals selectively influence the abundance of specific rhizosphere bacteria, indirectly broadening plant defense [56,57,58]. Building on these findings, we further explored the potential associations among rhizosphere microbes, metabolites, and immune-related activities in *G. elata*. In line with the conceptual framework of the plant immune system, our analyses suggest a multi-layered linkage between microbial taxa and immune-associated metabolic pathways and may serve as candidate “core microbial nodes” associated with immune-related metabolic processes. *Edaphobaculum* showed a positive association with 2-methoxyhydroquinone, a metabolite that significantly inhibits TNF-α and displays potency comparable to, or exceeding, previously reported plant-beneficial microbes [59], *Hypocrea* was correlated with both TNF-α inhibition and increased accumulation of flavonoids, suggesting its potential involvement in multiple immune-related metabolic pathways, although functional validation is still required. Notably, 2-methoxyhydroquinone was positively associated with both *Edaphobaculum* and *Hypocrea*, indicating that it may act as a key signaling metabolite within microbe-mediated immune modulation. This observation aligns with the emerging concept that certain metabolites can function simultaneously as signaling molecules and immunomodulatory mediators the fungal subnetworks [60], Cyphellophora displayed significant associations with 4-hydroxyphenylacetylglycine and a suite of anti-inflammatory peptides, suggesting that fungi may participate in more complex, potentially higher-order regulatory networks [61]. These findings indicate that fungi may occupy a central role in inflammation suppression and immune homeostasis by establishing high-dimensional immune regulatory networks, thereby expanding the current paradigm of fungus–metabolite–immune interactions. Importantly, our findings suggest that high-quality *G. elata* is characterized not simply by elevated levels of individual constituents but by a coordinated metabolic profile supported by a balanced rhizosphere microbial network and enhanced immunomodulatory metabolite pathways. These features jointly reflect a microbe–metabolite–activity equilibrium that underlies the formation of superior medicinal quality.

Overall, the synergistic action of these mechanisms promotes the accumulation and diversification of bioactive compounds in *G. elata*, thereby enhancing its medicinal quality and expanding its application potential. Nevertheless, this study has several limitations: the sampling area and sample size were relatively restricted, and the absence of standardized field blanks may limit the generalizability of our findings across different ecological conditions. Future work should broaden the geographic and sample coverage, adopt more rigorous experimental designs, and integrate microbial manipulation, targeted metabolite tracing, quantitative analyses of mycorrhizal interactions, and the isolation of mycorrhizal fungi to identify *G. elata*-specific microbial partners. Such efforts will provide a more robust theoretical foundation for improving the quality and industrial utilization of *G. elata.*

## 5. Conclusions

Overall, our study, by exploring the correlations between soil microbial communities, metabolomics, and pharmacological activity, provides evidence that the rhizospheric microbial network is associated with the pharmacological quality of *G. elata*. Our findings reveal that the bacterial community is primarily dominated by *Proteobacteria* and *Acidobacteria*, whereas the fungal community consists predominantly of *Ascomycota* and *Basidiomycota*. Significant functional differences exist between bacterial and fungal communities across the seven major planting areas. Bacterial community structure is predominantly influenced by chemical factors, notably pH and TN, whereas fungal communities exhibit unique sensitivity to altitude gradients. Furthermore, integrated analyses indicate significant correlations between secondary metabolites (including flavonoids and phenolic acids), anti-inflammatory bioactive compounds in tubers, and microorganisms such as *Edaphobaculum*, *Hypocrea*, *Cyphellophora*, *unidentified Cyanobacteria*, *Pseudomonas*, and Anaeromyxobacter. In summary, this research elucidates complex interactions among rhizosphere microorganisms, plant secondary metabolites, and biological activities of *G. elata*, thereby revealing microbial mechanisms underlying quality formation and providing novel theoretical perspectives for medicinal plant microbiome research and quality regulation strategies.

## Figures and Tables

**Table 1:** Climatic factors of sampling points in different producing areas.

	MXAT (°C)	MIAT (°C)	MMT (°C)	MMTD (°C)	APP (mm)	AAH (RH)	Altitude (m)	E	N
MB	24.30	11.16	16.00	20.25	1635.00	0.90	1493.00	98°40′	24°56′
DX	23.00	11.00	16.60	11.41	810.80	0.84	1779.00	104°15′	27°42′
XCB	18.00	7.50	12.75	10.18	631.00	0.68	1840.00	104°15′	27°46′
YS	22.00	12.00	15.00	10.10	1077.00	0.72	735.00	103°38′	28°16′
ZX	19.80	9.60	14.70	10.16	840.50	0.80	1682.00	104°52′	27°27′
LQ	22.00	11.00	16.50	12.75	664.10	0.70	1995.00	102°52′	25°54′
HZ	19.50	10.50	15.00	12.75	985.50	0.77	1659.00	104°53′	27°9′

**Table 2:** Chemical properties of rhizosphere soil for *G. elata*.

	MB	DX	XCB	YS	ZX	LQ	HZ	Significant
pH	4.97 ± 0.03 d	4.54 ± 0.03 f	4.7 ± 0.00 e	4.64 ± 0.015 e	6.27 ± 0.00 a	5.32 ± 0.02 c	5.44 ± 0.05 b	***
SOM	35.35 ± 0.17 c	41.24 ± 1.47 b	62.28 ± 1.24 a	24.58 ± 0.29 d	4.44 ± 0.14 f	21.54 ± 1.29 e	60.08 ± 1.29 a	***
TN	0.88 ± 0.02 de	1.24 ± 0.05 b	1.49 ± 0.05 a	1.07 ± 0.00 c	0.82 ± 0.09 e	0.95 ± 0.02 d	1.55 ± 0.02 a	***
TP	1.22 ± 0.02 a	0.77 ± 0.07 d	0.10 ± 0.04 c	1.04 ± 0.06 bc	1.09 ± 0.11 bc	1.14 ± 0.05 ab	0.84 ± 0.03 d	***
TK	1.15 ± 0.05 c	0.53 ± 0.02 d	2.43 ± 0.05 b	0.97 ± 0.04 c	0.65 ± 0.04 d	5.16 ± 0.40 a	0.53 ± 0.00 d	***
AP	21.68 ± 0.09 b	161.85 ± 1.48 a	12.87 ± 0.32 d	10.42 ± 0.30 e	20.30 ± 0.17 c	13.42 ± 0.22 d	9.21 ± 0.25 e	***
AK	4.51 ± 0.01 f	75.10 ± 1.02 b	114.02 ± 2.30 a	47.70 ± 0.25 d	43.22 ± 3.23 e	46.09 ± 0.57 de	51.56 ± 1.14 c	***
NO_3_^−^-N	3.33 ± 0.29 e	18.27 ± 1.42 a	7.45 ± 0.02 c	2.94 ± 0.18 e	7.12 ± 0.25 c	10.10 ± 0.64 b	4.77 ± 0.22 d	***
NH_4_^+^-N	58.68 ± 0.42 e	574.74 ± 9.34 b	931.60 ± 64.05 a	93.90 ± 2.08 de	302.00 ± 7.71 c	328.79 ± 1.55 c	108.87 ± 0.91 d	***

Values are presented as mean ± SE (*n* = 3). Different lowercase letters indicate statistically significant differences among treatments (***: *p* < 0.001).

## Data Availability

The raw 16S rRNA amplicon sequencing data have been deposited in the SRA database under the PRJNA1358749. The raw ITS amplicon sequencing data have been deposited in the NCBI Sequence Read Archive (SRA) under accession number PRJNA1358749. The raw UPLC–MS/MS metabolomics data have been deposited in the MassIVE repository under the accession number REQ20251110214503.

## Supplementary Materials

The following supporting information can be downloaded at: https://www.mdpi.com/article/10.3390/foods14244265/s1, Figure S1. Chemical composition of seven main producing areas of *G. elata*; Figure S2. Volcano diagram of differential metabolism of *G. elata* in seven producing areas (A: DX vs. MB; B: HZ vs. LQ; C: LQ vs. ZX; D: MB vs. HZ; E: XCB vs. DA; F: YS vs. XCB; G: ZX vs. YS); Figure S3. CCK-8 method was used to determine the activity of *G. elata* from different habitats on RAW264.7 cells; Figure S4. Bar chart of anti-inflammatory activity of *G. elata* crude extract (A: IL-6; B: NO; C: TNF-α). Control group vs. model group: *p* < 0.05; Model group vs. positive drug group: *p* < 0.05; Model group vs. drug group: *p* < 0.05. Celecoxib : 15 ug/mL, drug group: high concentration 1 mg/mL, medium concentration 0.5 mg/mL, low concentration 0.1 mg/mL; Figure S5. QC sample mass spectrometry detection TIC overlap diagram; Table S1. Metabolite Group Circular Diagram; Table S2. Statistical table of node degree of *G. elata* immune network in different producing areas; Table S3. CV-ANOVA verification of *G. elata* metabolomics from different producing areas; Table S4. Results of Tukey HSD Post Hoc Test for Shannon Diversity of Bacterial Communities in *G. elata* from Different Origins; Table S5. Results of Tukey HSD Post Hoc Test for Chao 1 Diversity of Bacterial Communities in *G. elata* from Different Origins.; Table S6. Results of Tukey HSD Post Hoc Test for Simpson Diversity of Bacterial Communities in *G. elata* from Different Origins; Table S7. Results of Tukey HSD Post Hoc Test for Unweighted UniFrac Distances of Bacterial Communities in *G. elata* from Different Origins; Table S8. Results of Tukey HSD Post Hoc Test for weighted UniFrac Distances of Bacterial Communities in *G. elata* from Different Origins; Table S9. Results of Tukey HSD Post Hoc Test for Fungal Chao1 Diversity in *G. elata* from Different Origins; Table S10. Results of Tukey HSD Post Hoc Test for Fungal Simpson Diversity in *G. elata* from Different Origins; Table S11. Results of Tukey HSD Post Hoc Test for Fungal Shannon Diversity in *G. elata* from Different Origins; Table S12. Results of Post Hoc Multiple Comparison Test for Unweighted UniFrac Distance of Fungal Communities in *G. elata* from Different Origins; Table S13. Results of Post Hoc Multiple Comparison Test for Weighted UniFrac Distance of Fungal Communities in *G. elata* from Different Origins.

## Abbreviations

The following abbreviations are used in this manuscript:

*Gastrodia elata* Bl	*G. elata*
National Medical Products Administration of China	NMPA
East longitude	E
North latitude	N
traditional Chinese medicinal	TCM
Tengchong City	MB
Ludian	DX
Yiliang	XCB
Yongshan	YS
Zhenxiong	ZX
Luquan	LQ
Hezhang	HZ
Cell Counting Kit-8	CCK-8
Cetyltrimethyl Ammonium Bromide	CTAB
annual maximum	MXAT
minimum temperatures	MIAT
annual mean temperature	MMT
mean monthly temperature difference	MMTD
annual precipitation	APP
annual average humidity	AAH
soil organic matter	SOM
total nitrogen	TN
total phosphorus	TP
total potassium	TK
available phosphorus	AP
available potassium	AK
nitrate nitrogen	NO_3_^−^-N
ammonium nitrogen	NH_4_^+^-N
temperature	T
Information-Dependent Acquisition	IDA
k-nearest neighbors	KNN
support vector regression	SVR
coefficient of variation	CV
enzyme-linked immunosorbent assay	ELISA
one-way analysis of variance	ANOVA
Bray–Curtis	BC
weighted UniFrac	WU
unweighted UniFrac	UU
