# Supplementary material for: Environmental and Rhizosphere Microbiome Drivers of Metabolic Profiles in Gastrodia elata: An Integrative Analysis of Soil, Metabolomics and Anti-Inflammatory Readouts
Source: Foods. 2025 Dec 11;14(24):4265. doi: 10.3390/foods14244265 (PMC12731933; doi:10.3390/foods14244265)
Supplement: Supplementary file 1 [file foods-14-04265-s001.zip › supplementary materials.pdf]

Supplementary Materials:

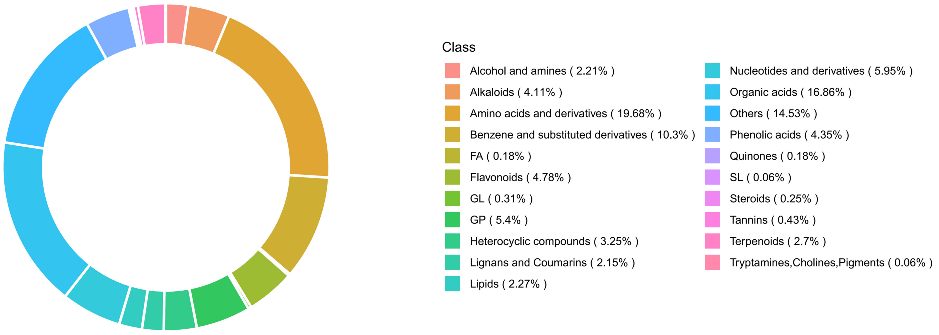

Figure S1. Chemical composition of seven main producing areas of *G. elata*

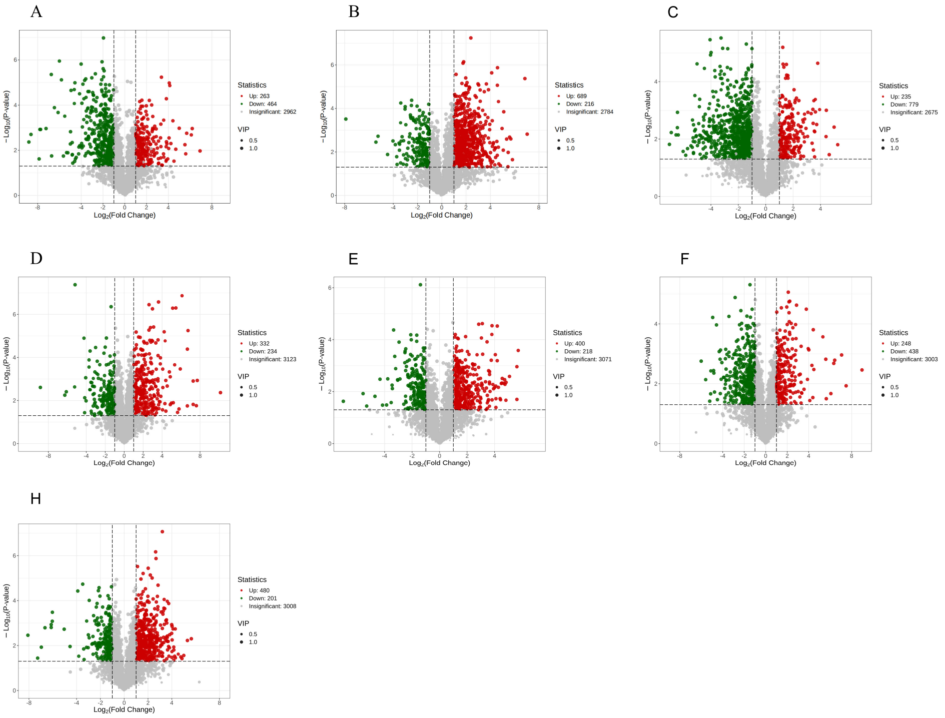

Figure S2. Volcano diagram of differential metabolism of *G. elata* in seven producing areas (A : DX vs MB ; B : HZ vs LQ ; C : LQ vs ZX ; D : MB vs HZ ; E : XCB vs DA ; F : YS vs XCB ; G : ZX vs YS)

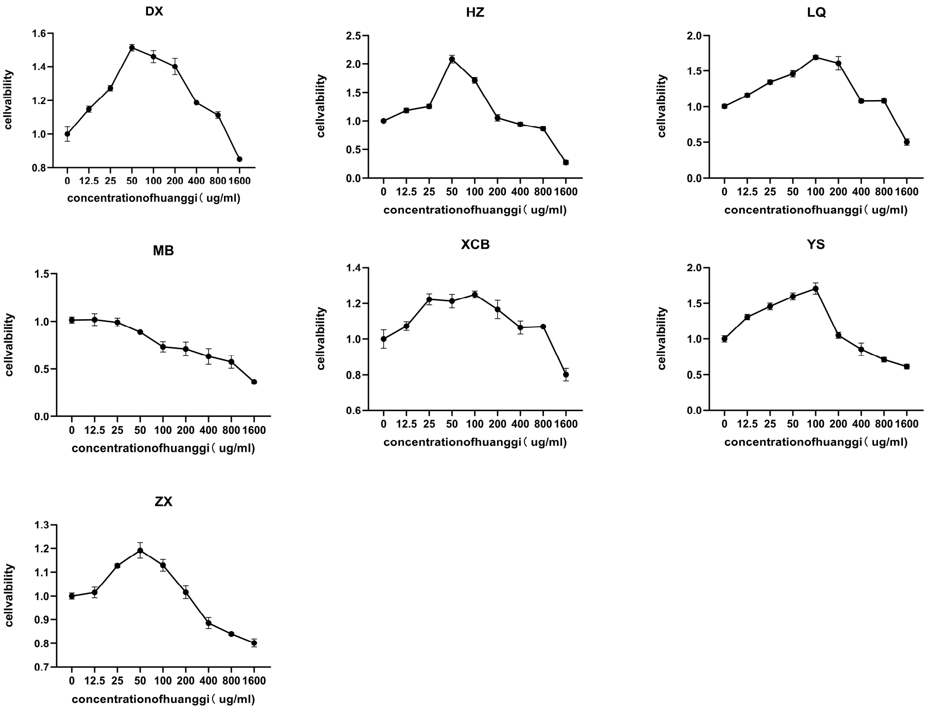

Figure S3. CCK-8 method was used to determine the activity of *G. elata* from different habitats on RAW264.7 cells.

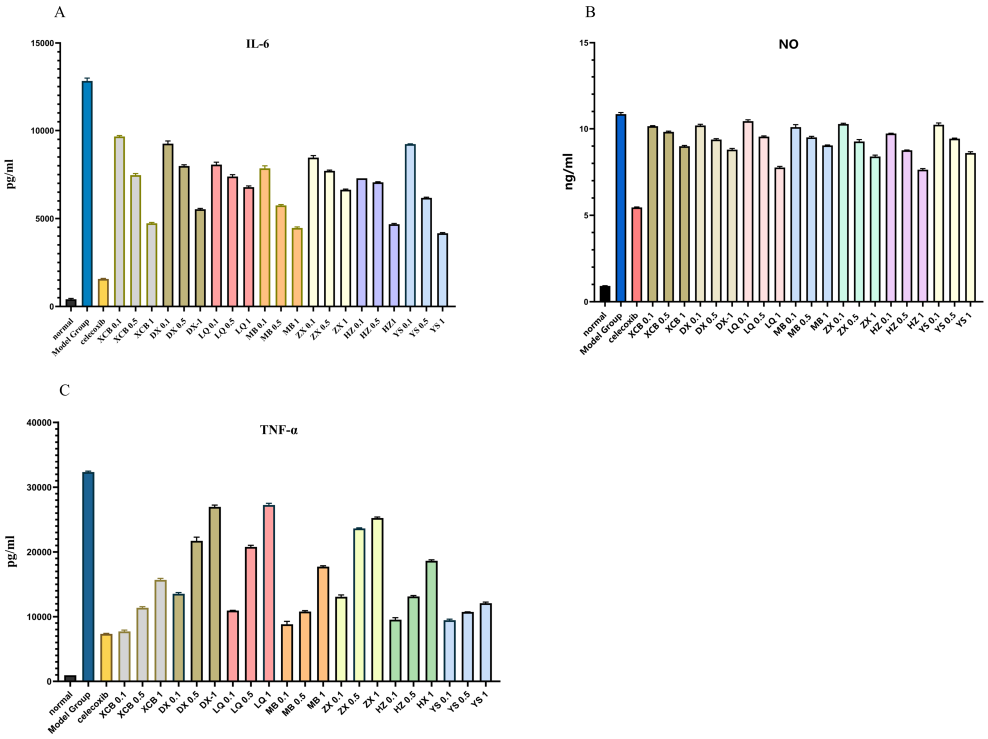

Figure S4. Bar chart of anti-inflammatory activity of *G. elata* crude extract (A: IL-6; B: NO; C: TNF-α). Control group vs model group: $P < 0.05$; Model group vs positive drug group: $P < 0.05$; Model group vs drug group: $P < 0.05$. Celecoxib : 15ug / ml, drug group: high concentration 1mg / ml, medium

concentration 0.5mg / ml, low concentration 0.1mg / ml.

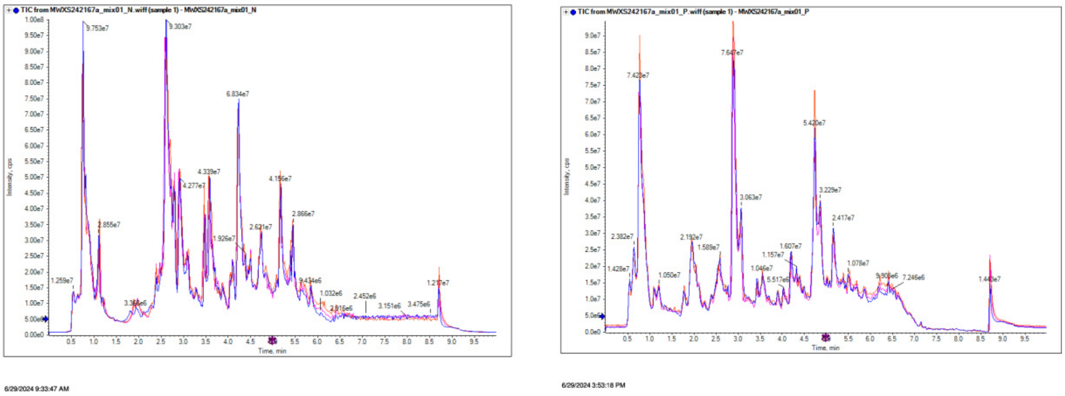

Figure S5. QC sample mass spectrometry detection TIC overlap diagram

Table S1. Metabolite Group Circular Diagram

Class.I	MB	DX	XCB	YS	ZX	LQ	HZ
Amino acids and derivatives	32.147	31.371	35.763	29.545	31.197	35.617	31.201
Others	21.189	20.524	19.340	22.540	20.528	18.344	22.167
Organic acids	11.483	12.130	9.641	12.206	11.278	10.345	11.693
Benzene and substituted derivatives	5.824	5.020	5.655	5.419	6.110	5.239	5.119
Phenolic acids	5.773	6.618	5.639	6.755	5.750	5.843	6.499
GL	4.629	4.713	4.615	4.738	4.764	5.413	4.996
Nucleotides and derivatives	3.799	3.413	3.738	3.602	3.825	2.755	3.635
Alkaloids	2.670	2.852	2.931	2.476	2.934	3.180	2.499
Alcohol and amines	2.535	2.688	2.389	2.391	2.718	2.814	2.431
Flavonoids	2.187	2.859	2.868	2.857	2.903	2.114	2.453
GP	1.856	2.069	1.687	2.031	1.918	2.282	2.056
Lignans and Coumarins	1.683	0.970	1.037	0.893	1.089	0.846	0.833
Lipids	1.598	1.496	2.123	1.523	1.929	2.128	1.600
Heterocyclic compounds	1.203	1.433	1.119	1.408	1.286	1.394	1.337
Terpenoids	0.498	0.567	0.479	0.507	0.551	0.557	0.532
Tryptamines,Cholines,Pigments	0.371	0.552	0.373	0.435	0.549	0.437	0.292
FA	0.287	0.368	0.347	0.345	0.368	0.391	0.353
Quinones	0.091	0.114	0.095	0.121	0.112	0.082	0.093
Steroids	0.085	0.132	0.080	0.108	0.090	0.117	0.116
SL	0.067	0.078	0.049	0.067	0.064	0.067	0.062
Tannins	0.024	0.032	0.031	0.031	0.039	0.036	0.032
	100	100	100	100	100	100	100

Table S2. Statistical table of node degree of *G. elata* immune network in different producing areas

name	BetweennessCentrality	ClosenessCentrality	Degree
g__Hypocrea	0.2147	0.5800	29
g__unclassified_Coniochaetales	0.2083	0.5918	29
g__Colletotrichum	0.1033	0.5088	21
g__Monographella	0.0500	0.4603	17
g__Codinaeopsis	0.1640	0.4265	17
g__Cyphellophora	0.0414	0.4496	16
FDATN00296	0.0559	0.4874	14
MW0133358	0.0559	0.4874	14
MW0148113	0.0559	0.4874	14
g__Chaetomium	0.0229	0.4328	13
g__Gliocladiopsis	0.0385	0.4173	13
TNF-α	0.0452	0.4640	10
MW0142257	0.0270	0.4677	10
MW0137262	0.0238	0.4567	10
MEDN1233	0.0213	0.4567	9

MW0129511	0.0164	0.4496	9
MW0134901	0.0142	0.4496	8
MW0003179	0.0142	0.4496	8
g__Lecythophora	0.0053	0.3742	8
g__Boothiomyces	0.0040	0.3537	7
g__Cephalotheca	0.0088	0.3766	7
MW0146178	0.0294	0.4715	7
MW0107255	0.0294	0.4715	7
g__Cyberlindnera	0.0040	0.3537	7
MW0115112	0.0214	0.4715	7
MW0105085	0.0073	0.3946	7
MW0150200	0.0214	0.4715	7
MW0009307	0.0073	0.3946	7
NO	0.0358	0.3791	6
g__Gibberella	0.0029	0.3580	6
g__Pochonia	0.0018	0.3452	5
MW0129333	0.0192	0.4531	5
MW0145968	0.0189	0.4462	5
MW0129711	0.0101	0.4567	5
MW0153891	0.0101	0.4567	5
MW0104606	0.0065	0.4234	5
MW0146184	0.0065	0.4234	5
g__Peziza	0.0015	0.3602	5
MW0129515	0.0411	0.4361	5
MW0148157	0.0034	0.4265	5
MW0146333	0.0034	0.4265	5
MW0015923	0.0034	0.4265	5
MEDP1548	0.0023	0.4113	4
g__Pyrenochaetopsis	0.0005	0.3412	4
g__Conlarium	0.0001	0.3372	3
MW0135445	0.0047	0.4234	3
MW0137162	0.0047	0.4234	3
MW0123675	0.0047	0.4234	3
MW0146059	0.0047	0.4234	3
MW0158878	0.7000	0.6250	3
MW0152463	0.0008	0.3946	3
IL6	0.4000	0.5000	2
MW0144494	0.0003	0.3841	2
MW0006988	0.0345	0.3037	2
MW0133725	0.0021	0.4000	2
g__Mollisia	0.6000	0.6250	2
g__unclassified_Archaeosporales	0.0000	0.3571	1
g__Ambispora	0.0000	0.3187	1
g__Physisporinus	0.0000	0.2762	1

g__unclassified_Fungi	0.0000	0.4167	1
g__Coprinellus	0.0000	0.2339	1
MW0118638	0.0000	0.3005	1
MW0109000	0.0000	0.3005	1
g__Leptodontidium	0.0000	0.3053	1
g__Cladophialophora	0.0000	0.4167	1

Table S3. CV-ANOVA verification of *G. elata* metabolomics from different producing areas

group	SS	DF	MS	F	p	SD
DX_vs_MB	4.94773	3	1.64924	63.1055	0.0156398	1.28423
HZ_vs_LQ	4.97	3	1.65667	110.45	0.00898601	1.28712
LQ_vs_ZX	4.93792	3	1.64597	53.029	0.0185655	1.28296
MB_vs_HZ	4.94359	3	1.64786	58.4282	0.0168741	1.28369
XCB_vs_DX	4.87318	3	1.62439	25.6181	0.0378026	1.27452
YS_vs_XCB	4.90975	3	1.63658	36.2679	0.0269524	1.27929
ZX_vs_YS	4.87384	3	1.62461	25.7538	0.0376097	1.2746

Table S4. Results of Tukey HSD Post Hoc Test for Shannon Diversity of Bacterial Communities in *G. elata* from Different Origins.

Group	Difference	P-value	Signif	Lower_limit	Upper_limit
HZ - LQ	12.66666667	0	***	8.210121805	17.12321153
HZ - MB	13.33333333	0	***	8.876788471	17.7898782
LQ - ZX	-15.66666667	0	***	-20.12321153	-11.2101218
YS - ZX	-6.666666667	0.0063	**	-11.12321153	-2.210121805
DX - MB	6	0.0119	*	1.543455138	10.45654486
XCB - YS	-4.333333333	0.0558	ns	-8.789878195	0.123211529
DX - XCB	0.666666667	0.7531	ns	-3.789878195	5.123211529

Table S5. Results of Tukey HSD Post Hoc Test for Chao 1 Diversity of Bacterial Communities in *G. elata* from Different Origins.

Group	Difference	P-value	Signif	Lower_limit	Upper_limit
HZ - MB	12.66666667	0.0127	*	3.158995494	22.17433784
HZ - LQ	10	0.0406	*	0.492328827	19.50767117
LQ - ZX	-6.333333333	0.175	ns	-15.84100451	3.17433784
YS - ZX	-6.333333333	0.175	ns	-15.84100451	3.17433784
XCB - YS	5.666666667	0.2219	ns	-3.841004506	15.17433784
DX - MB	4.333333333	0.3449	ns	-5.17433784	13.84100451
DX - XCB	-4	0.3821	ns	-13.50767117	5.507671173

Table S6. Results of Tukey HSD Post Hoc Test for Simpson Diversity of Bacterial Communities in *G. elata* from Different Origins.

Group	Difference	P-value	Signif	Lower_limit	Upper_limit
LQ - ZX	-13.66666667	0.0001	***	-19.18466873	-8.148664608
XCB - YS	-8.5	0.0052	**	-14.01800206	-2.981997941
HZ - LQ	7.166666667	0.0146	*	1.648664608	12.68466873
HZ - MB	5.666666667	0.0449	*	0.148664608	11.18466873
DX - XCB	-3.166666667	0.2387	ns	-8.684668726	2.351335392
DX - MB	0.5	0.8487	ns	-5.018002059	6.018002059
YS - ZX	0	1	ns	-5.518002059	5.518002059

Table S7. Results of Tukey HSD Post Hoc Test for Unweighted UniFrac Distances of Bacterial Communities in *G. elata* from Different Origins.

Group	Difference	P-value	Signif	Lower_limit	Upper_limit
DX - MB	13.66666667	0	***	9.807185603	17.52614773
LQ - ZX	-9.666666667	0.0001	***	-13.52614773	-5.807185603
XCB - YS	9.333333333	0.0001	***	5.47385227	13.1928144
HZ - LQ	6.333333333	0.0034	**	2.47385227	10.1928144
HZ - MB	6	0.0049	**	2.140518936	9.859481064
YS - ZX	-2.666666667	0.1605	ns	-6.52614773	1.192814397
DX - XCB	-2.333333333	0.2157	ns	-6.192814397	1.52614773

Table S8. Results of Tukey HSD Post Hoc Test for weighted UniFrac Distances of Bacterial Communities in *G. elata* from Different Origins.

Group	Difference	P-value	Signif	Lower_limit	Upper_limit
YS - ZX	11	0.0095	**	3.140425911	18.85957409
HZ - MB	-9.333333333	0.0233	*	-17.19290742	-1.473759244
DX - XCB	2	0.5938	ns	-5.859574089	9.859574089
LQ - ZX	2	0.5938	ns	-5.859574089	9.859574089
XCB - YS	-1.333333333	0.7214	ns	-9.192907423	6.526240756
HZ - LQ	0.666666667	0.8582	ns	-7.192907423	8.526240756
DX - MB	-0.333333333	0.9288	ns	-8.192907423	7.526240756

Table S9. Results of Tukey HSD Post Hoc Test for Fungal Chao1 Diversity in *G. elata* from Different Origins.

Group	Difference	P-value	Signif	Lower_limit	Upper_limit
HZ - LQ	15.66666667	0	***	12.27008255	19.06325078
HZ - MB	16	0	***	12.60341588	19.39658412
LQ - ZX	-10.66666667	0	***	-14.06325078	-7.270082552
XCB - YS	8.666666667	0.0001	***	5.270082552	12.06325078
DX - MB	7	0.0006	***	3.603415885	10.39658412
DX - XCB	-7	0.0006	***	-10.39658412	-3.603415885
YS - ZX	-5.666666667	0.003	**	-9.063250782	-2.270082552

Table S10. Results of Tukey HSD Post Hoc Test for Fungal Simpson Diversity in *G. elata* from Different Origins.

Group	Difference	P-value	Signif	Lower_limit	Upper_limit
LQ - ZX	-15.5	0	***	-21.01138178	-9.988618222
YS - ZX	-13.16666667	0.0002	***	-18.67804844	-7.655284888
XCB - YS	11.5	0.0005	***	5.988618222	17.01138178
HZ - LQ	8.833333333	0.004	**	3.321951555	14.34471511
DX - XCB	-7	0.0165	*	-12.51138178	-1.488618222
HZ - MB	6	0.035	*	0.488618222	11.51138178
DX - MB	4	0.1419	ns	-1.511381778	9.511381778

Table S11. Results of Tukey HSD Post Hoc Test for Fungal Shannon Diversity in *G. elata* from Different Origins.

Group	Difference	P-value	Signif	Lower_limit	Upper_limit
LQ - ZX	-13	0	***	-17.11583374	-8.884166259
XCB - YS	13.33333333	0	***	9.217499592	17.44916707
YS - ZX	-12.33333333	0	***	-16.44916707	-8.217499592
HZ - MB	9.666666667	0.0002	***	5.550832925	13.78250041
HZ - LQ	9	0.0003	***	4.884166259	13.11583374
DX - XCB	-8	0.0009	***	-12.11583374	-3.884166259
DX - MB	6.666666667	0.0037	**	2.550832925	10.78250041

Table S12. Results of Post Hoc Multiple Comparison Test for Unweighted UniFrac Distance of Fungal Communities in *G. elata* from Different Origins.

Group	Difference	P-value	Signif	Lower_limit	Upper_limit
LQ - ZX	-14	0.0001	***	-19.62935576	-8.370644244
DX - MB	-12.66666667	0.0003	***	-18.29602242	-7.03731091
HZ - MB	-8.333333333	0.0067	**	-13.96268909	-2.703977577
HZ - LQ	7.333333333	0.0143	*	1.703977577	12.96268909
DX - XCB	-6.666666667	0.0236	*	-12.29602242	-1.03731091
XCB - YS	-3.666666667	0.1842	ns	-9.296022423	1.96268909
YS - ZX	-0.666666667	0.8032	ns	-6.296022423	4.96268909

Table S13. Results of Post Hoc Multiple Comparison Test for Weighted UniFrac Distance of Fungal Communities in *G. elata* from Different Origins.

Group	Difference	P-value	Signif	Lower_limit	Upper_limit
DX - MB	-16.66666667	0	***	-21.45493692	-11.87839641
HZ - MB	-13.33333333	0	***	-18.12160359	-8.545063075
HZ - LQ	-11	0.0002	***	-15.78827026	-6.211729742
LQ - ZX	5.333333333	0.0315	*	0.545063075	10.12160359
XCB - YS	-5.333333333	0.0315	*	-10.12160359	-0.545063075
DX - XCB	-3.666666667	0.1228	ns	-8.454936925	1.121603592
YS - ZX	0	1	ns	-4.788270258	4.788270258
